# Dominant plant species play an important role in regulating bacterial antagonism in terrestrial Antarctica

**DOI:** 10.3389/fmicb.2023.1130321

**Published:** 2023-03-23

**Authors:** Beenish Naz, Ziyang Liu, Lucie A. Malard, Izhar Ali, Hongxian Song, Yajun Wang, Xin Li, Muhammad Usman, Ikram Ali, Kun Liu, Lizhe An, Sa Xiao, Shuyan Chen

**Affiliations:** ^1^Ministry of Education Key Laboratory of Cell Activities and Stress Adaptations, School of Life Sciences, Lanzhou University, Lanzhou, Gansu, China; ^2^Department of Ecology and Evolution, University of Lausanne, Lausanne, Switzerland; ^3^College of Pastoral Agriculture Science and Technology, Lanzhou University, Lanzhou, Gansu, China; ^4^State Key Laboratory of Herbage Improvement and Grassland Agro-ecosystems, College of Ecology, Lanzhou University, Lanzhou, Gansu, China

**Keywords:** Antarctica, bacterial antagonism, dominant plants, edaphic characteristics, structural equation model

## Abstract

In Antarctic terrestrial ecosystems, dominant plant species (grasses and mosses) and soil physicochemical properties have a significant influence on soil microbial communities. However, the effects of dominant plants on bacterial antagonistic interactions in Antarctica remain unclear. We hypothesized that dominant plant species can affect bacterial antagonistic interactions directly and indirectly by inducing alterations in soil physicochemical properties and bacterial abundance. We collected soil samples from two typical dominant plant species; the Antarctic grass *Deschampsia antarctica* and the Antarctic moss *Sanionia uncinata,* as well as bulk soil sample, devoid of vegetation. We evaluated bacterial antagonistic interactions, focusing on species from the genera *Actinomyces*, *Bacillus*, and *Pseudomonas*. We also measured soil physicochemical properties and evaluated bacterial abundance and diversity using high-throughput sequencing. Our results suggested that Antarctic dominant plants significantly influenced bacterial antagonistic interactions compared to bulk soils. Using structural equation modelling (SEM), we compared and analyzed the direct effect of grasses and mosses on bacterial antagonistic interactions and the indirect effects through changes in edaphic properties and bacterial abundance. SEMs showed that (1) grasses and mosses had a significant direct influence on bacterial antagonistic interactions; (2) grasses had a strong influence on soil water content, pH, and abundances of *Actinomyces* and *Pseudomonas* and (3) mosses influenced bacterial antagonistic interactions by impacting abundances of *Actinomyces*, *Bacillus*, and *Pseudomonas*. This study highlights the role of dominant plants in modulating bacterial antagonistic interactions in Antarctic terrestrial ecosystems.

## Introduction

Antarctica is the coldest, driest, and windiest continent and is considered one of the harshest environments for biodiversity (Chown et al., 2015; Wong et al., 2019), yet it harbors surprisingly complex communities with high bacterial diversity (Smith et al., 2010). The diversity and composition of soil microbiomes in Antarctica have been documented in several studies (O’Brien et al., 2004; Wong et al., 2011; Bottos et al., 2014; Peixoto et al., 2016; Malard and Pearce, 2018; Malcheva et al., 2020; Zhang et al., 2020) which have shown that bacterial communities across Antarctic soils vary significantly not only with climate, geography and local biological influences, but also with plant type (Griffiths et al., 2011; Xiong et al., 2012; Yun et al., 2014). Indeed, plants affect soil microbial community composition (Kowalchuk et al., 2002), diversity, and microbial interactions (Wang et al., 2022) by providing nutrients (Anderson, 2011; Schlatter et al., 2015) and inducing competition for resources (Rousk et al., 2010). For instance, research has shown that dominant plants enhance microbial abundance and soil resources (carbon, nitrogen, and phosphorus) when compared to Antarctic bulk soil (Benavent-González et al., 2018). Furthermore, varying composition of exudates may alter the abundance, composition, and activity of soil microorganisms (Chung et al., 2007; Liu et al., 2008), thereby affecting edaphic properties such as soil water content, pH, and nutrient availability (Wang et al., 2020; Wan et al., 2021) and impacting microbial interactions (Wang et al., 2022).

Microbial interactions within the soil community primarily involve antagonistic interactions (Hall et al., 2020). The phenomenon in which one organism inhibits the growth or development of other microorganisms is called antagonism (Chou et al., 2009) and is commonly observed within soil bacterial communities (Kinkel et al., 2012; Schlatter and Kinkel, 2014; Essarioui et al., 2017). Plants have been suggested as the main factors responsible for the establishment of antagonistic bacteria in the rhizosphere (Latz et al., 2012). Indeed, by selecting specific antagonistic phenotypes and changing the gene expression of soil microorganisms, plants can actively engage in co-evolving antagonistic interactions with their soil bacteria (Kinkel et al., 2011). The development and maintenance of microbial populations depends on such antagonism, which also serves to control various plant diseases (Chou et al., 2009). For example, many rhizosphere bacteria produce antibiotics that inhibit soil borne pathogens, improving plant growth under pathogen pressure, and enhancing primary productivity in terrestrial ecosystems (Haas and Keel, 2003). However, it remains difficult to extensively examine microbial antagonism or competitive interactions *in situ*.

Most of our knowledge on the roles of antibiotics in mediating microbial antagonistic interactions comes from studies on the bacterial domain. For example, members of the genus *Actinomyces* (*Actinobacteria*) are considered the most prolific producers of secondary metabolites, including antifungals, antivirals, and antibiotics (de Lima Procópio et al., 2012). *Bacillus* (*Firmicutes*) is also one of the most common and functionally diverse bacterial genera of the soil. Members of this genus are well known for their capacity to produce antimicrobial compounds, participate in the cycling of nutrients, and protect plants from pathogens in different ecosystems (Saxena et al., 2020). Similarly, *Pseudomonas* (*Gammaproteobacteria*) species can be involved in antagonistic activities, especially antifungal activity in soil (Becker et al., 2012; Latz et al., 2012, 2016). Studies have shown that plant species and plant productivity can affect microbial antagonism mediated by *Actinomyces* (Bakker et al., 2014; Schlatter et al., 2015), *Bacillus* (Latz et al., 2016), and *Pseudomonas* (Becker et al., 2012; Latz et al., 2012, 2015). However, the impact of dominant plants on these antagonistic bacterial communities and their interactions in Antarctic soils remains unclear despite microbial co-evolutionary interactions being crucial for ecosystem functionality (Schlatter et al., 2019) and biodiversity (Becker et al., 2012).

In this study, we analyzed the effects of Antarctic dominant plant species on the bacterial antagonistic potential on the Fildes Peninsula (King George Island). We compared a dominant flowering plant species, the Antarctic hair grass *Deschampsia antarctica,* with a non-flowering moss species *Sanionia uncinata* (Melick and Seppelt, 1997; Benavent-González et al., 2018). The primary aims were to (1) analyze the direct effects of Antarctic dominant plants on the bacterial antagonistic potential and their indirect effects through changes in soil properties and bacterial abundance; and (2) to evaluate the cultivable densities and antagonist densities of *Actinomyces*, *Bacillus* and *Pseudomonas* under dominant plants and in bulk soils. We specifically targeted *Actinomyces*, *Bacillus* and *Pseudomonas* to evaluate antagonism because of their ubiquitous presence in the vegetated soils of polar regions (Teixeira et al., 2010; Peixoto et al., 2016; Poosakkannu et al., 2017; Malard and Pearce, 2018; Malard et al., 2019), and their recognized ability for producing a wide variety of antagonistic compounds (Raaijmakers and Mazzola, 2012).

## Materials and methods

### Sampling site and experimental design

The study was conducted on the Fildes Peninsula (62°08′ to 62°14′S and 59°02′ to 58°51′W), near the Great Wall Chinese research station, southwest of King George Island, Antarctica, during the austral summer of 2019–2020. To determine the direct and indirect effects of Antarctic dominant plants on antagonistic bacterial communities, we collected soil samples with and without the dominant plants (*Deschampsia antarctica* and *Sanionia uncinata*). To do so, we selected 3 experimental sites dominated by the abovementioned plant species. At each site, we randomly established five (10 m × 10 m) plots with more than 10 plant individuals. Within each (10 m × 10 m) plot, three (30 cm × 30 cm) quadrats were established: one quadrat was placed around an adult moss individual, one quadrat was placed around an adult grass individual and one quadrat was placed on bare soil. 200 g of soil was collected from each quadrat [3 sites × 5 plots × (2 plants + 1 bulk soil quadrats)], close to the center of the base of plants (topsoil, sampling depth 0 to 10 cm). To ensure that all plots shared the same climatic conditions, there was less than a 10 m distance between experimental sites. Each soil sample was divided into three equal-sized aliquots for (1) the analysis of soil physicochemical properties, (2) DNA sequencing, and (3) the characterization of bacterial antagonistic potential (Supplementary Figure S1).

### Edaphic properties

Each soil sample was assessed for the determination of soil properties according to routine procedures. 10 g of soil was dried for 72 h at 105°C to determine the moisture content (SWC) of the soil (Yang et al., 2021). Then, the soil was sieved using a 100 mesh (0.15 mm) and analyzed for total phosphorus (TP), total nitrogen (TN) and soil organic carbon (SOC), ammonium (NH_4_^+^), nitrates (NO_3_^−^), and pH. Total nitrogen and total phosphorus were digested by concentrated H_2_SO_4_ and measured by semi-micro Kjeldahl and Mo-Sb antispetrophotography (Tian et al., 2017), respectively, using an auto chemistry analyzer (Smart Chem 200, AMS Alliance, United States). Soil organic carbon was measured based on the wet oxidation method (Wang et al., 2022). Both ammonium and nitrate were determined after extracting with 2 M KCl (Cui et al., 2022). A pH meter was used to measure the pH of the soil in a 1:1.5 soil water slurry (PHSJ-3F, Shanghai INESA Scientific Instrument Co., Ltd., China).

### Soil DNA extraction and sequencing

Genomic DNA was extracted using the DNeasy PowerSoil^®^ Kit (Qiagen, Germany) following the manufacturer’s protocol. For the composition of the bacterial community, the V4 region of bacterial 16S rRNA genes was amplified using specific primers: 515F (GTGYCAGCMGCCGCGGTAA) and 806R (GGACTACNVGGGTWTCTAAT). The PCR products were visualized using a 2% agarose gel. Then, the Qiagen Gel Extraction Kit (Qiagen, Germany) was used to purify the PCR product mixture. The TruSeq^®^ DNA PCR-Free Sample Preparation Kit (Illumina, United States) was used to prepare the sequencing libraries. The library quality was assessed on the Qubit@2.0 Fluorometer (ThermoFisher Scientific, United States) and Agilent Bioanalyzer 2,100 system (Agilent, United States). The library was sequenced on an Illumina NovaSeq platform (Illumina, United States) using PE250 strategy. Artificial sequences and low-quality sequences were removed using the fastp software (Chen et al., 2018). We obtained a total of 6,781,628 bacterial sequences, of which 2,690,280 quality-screened sequences belonged to our phyla of interest: *Actinobacteria*, *Firmicutes* and *Proteobacteria*. Operational taxonomic units (OTUs) at 99% similarity were generated using the dada2-paired pipeline integrated to the QIIME2 software (QIIME2.2021.2).

### Quantification of antagonistic bacteria

The soil aliquots designated for the antagonistic potential investigation were further divided into three equal-sized aliquots for a total of 135 soil samples for bacterial antagonistic evaluation. Samples were kept at 4°C and each soil sample was evaluated for cultivable bacterial densities. Three cultivable bacterial groups were quantified, comprising members of the genera *Actinomyces*, *Bacillus*, and *Pseudomonas*. For simplicity, we use the term ‘bacterial’ with abundance, diversity and antagonism in many places throughout the manuscript to refer to these groups. We used the dilution method of Latz et al. (2016) (with minor modifications) to measure indices of bacterial antagonistic interactions (total cultivable densities, antagonist densities, inhibition intensities and frequencies). For each sample, 5 g of soil was suspended in 50 ml phosphate-buffered saline (PBS) and shaken at 4°C (*Actinomyces*: 175 rpm for 60 min; *Bacillus* and *Pseudomonas*: 200 rpm for 120 min). For the enumeration of *Actinomyces*, 100 μl of each soil dilution was plated on starch-casein agar containing 100 μg ml^−1^ of cycloheximide and incubated for 7 days at 20°C. For *Bacillus*, the soil suspension was first incubated at 85°C for 30 min. Dilutions of the suspension were then plated on Tryptic soy agar and incubated for 4 days at 20°C. Finally, for *Pseudomonas*, the dilutions were plated on King’s B agar containing 100 μg ml^−1^ of cycloheximide, 13 μg ml^−1^ of chloramphenicol, and 40 μg ml^−1^ of ampicillin, and incubated for 5 days at 20°C.

In natural ecosystems, fungal pathogens represent essential components of biodiversity (Benítez-Malvido et al., 2021), hence we used two fungal pathogens and a bacterial antagonist to assess bacterial antagonistic potential. The three target strains used were *Fusarium graminearum* (ACCC 36249), *Rhizoctonia solani* (strain 3.1704) and *Streptomyces fildesensis* (strain INACH3013) (Núñez-Montero et al., 2019). *Fusarium* species (Ascomycota) and *Rhizoctonia* species (Basidiomycota) are ubiquitously present in Antarctic soils, particularly in King George Island (Durán et al., 2019), while the selected *Streptomyces* strain is an indigenous Antarctic isolate. We followed the overlay method of Bakker et al. (2010) by pouring 14 ml of the second medium layer (detailed hereafter) over the enumeration plates previously described. For *Fusarium*, the complete content of an oatmeal agar (OA) Petri dish, incubated at room temperature for 7 days was homogenized (for a few seconds) with 100 ml of distilled water in a sterile blender. Then, 500 ml of molten potato dextrose agar (PDA), cooled to 45°C, was inoculated with 20 ml of the *Fusarium* solution obtained and poured over the enumeration plates. The plates were incubated at 25°C for 4 days. For *Rhizoctonia*, liquid cultures in Czapek-Dox (CD) broth (incubated for 7 days at room temperature) were homogenized in a sterile blender (for a few seconds). 100 ml of this *Rhizoctonia* solution was added per 500 ml of molten CD agar (1% final concentration agar, cooled to 45°C). This medium was poured over the enumeration plates and incubated at room temperature (25°C) for 4–5 days. For *Streptomyces*, 150 μl of a 20% glycerol suspension containing approximately 10^8^ CFU/ml of *Streptomyces fildesensis* was plated over the enumeration plates and incubated at 28°C for 5 days (optimum growth temperature was determined in pilot experiments) (Wiggins and Kinkel, 2005). Three soil dilution plates were spread per inhibitory strain.

The antagonistic potential for all samples [(135 soil samples × 3 inhibitory strains × 3 dilutions) = 1,215] was evaluated. The radius of the inhibition zone was used to assess antagonism (Bakker et al., 2010; Ji et al., 2014). For each isolate, the average of the inhibitory intensity, antagonist density, and frequency values was calculated for each sample (Bakker et al., 2013).

### Statistical analysis

Data analysis and plotting were conducted in R, version 4.0.2 (CoreTEAM R, 2020). Statistical differences of the indices of antagonistic interactions between treatments (vegetated/non-vegetated soils) were tested using Tukey’s HSD test. A principal component analysis (PCA) was conducted to evaluate and visualize the effect of dominant plants on bacterial antagonism using permutational multivariate analysis of variance (PERMANOVA). Focusing on the phyla of interest, principal co-ordinate analyses (PCoA) were conducted to quantify compositional differences between samples based on Bray–Curtis dissimilarity matrices, using bacterial OTUs at the genus level. A redundancy analysis (RDA) was carried out to analyze the influence of dominant plants and edaphic properties on the bacterial communities. A Pearson’s correlation analysis was carried out to provide insight into the selection processes affecting bacterial antagonistic characteristics (Bakker et al., 2013). A heatmap was constructed to display the relationships between the indices of antagonistic interactions, edaphic properties, and the relative abundances of bacterial taxa.

We used structural equation modelling (SEM) analyses in accordance with the following assumptions: we hypothesized that (1) dominant plants have a significant influence on the antagonistic bacterial potential of *Actinomyces*, *Bacillus* and *Pseudomonas* (Wang et al., 2022); (2) Antarctic dominant plants induce changes in soil physicochemical properties (Benavent-González et al., 2018); (3) bacterial abundance in vegetated soil (grass and moss associated soil) is correlated with bacterial antagonistic interactions (Benavent-González et al., 2018); (4) plant type (flowering and non-flowering) is linked with bacterial antagonistic potential; (5) Soil bacterial antagonistic interactions can be shaped by dominant plant species in the Antarctic. First, our conceptual model assumed pathways linking dominant plants and antagonistic interactions *via* edaphic properties and bacterial communities (Supplementary Figure S2; Supplementary Table S1). The effects on bacterial antagonistic potential were the main focus of both SEMs (one for each type of plant). We used PC1 values (i.e., the first axis of Principal component analysis) of the antagonistic activities of *Actinomyces*, *Bacillus*, and *Pseudomonas* to represent bacterial antagonism altogether by applying the method to evaluate the direct and indirect effect of grasses and mosses on the antagonistic interactions of *Actinomyces*, *Bacillus* and *Pseudomonas* (Veen et al., 2010).

There are several indices that evaluate the goodness of fit for structural equation models; however, no single index is approved universally. Therefore, to evaluate the accuracy of SEM, we combined the root mean square error of approximation (RMSEA) test and χ2 test. A good model fit to the data was indicated by a non-significant χ2 and RMSEA test (Kline, 2011). The goodness of the model fit was also evaluated through the goodness of fit index (GFI > 0.90 indicates a good fit) value (Kline, 2005). Using the ‘car’ package, the variance homogeneity was evaluated (Fox, 2011), the correlations were performed using the ‘psych’ package (Revelle, 2018), and the ‘lmPerm’ package was used to carry out the permutation test (Wheeler and Torchiano, 2016). The ‘vegan’ package was used to perform RDA and PCA (Oksanen et al., 2014). The ‘lavaan’ package (Rosseel, 2012) was used to conduct the SEMs, and the ‘ggplot2’ package was used to plot the figures (Wickham, 2010).

## Results

### Dominant plant effects on *Actinomyces*, *Bacillus*, and *Pseudomonas* antagonism indices

**Figure 1:**
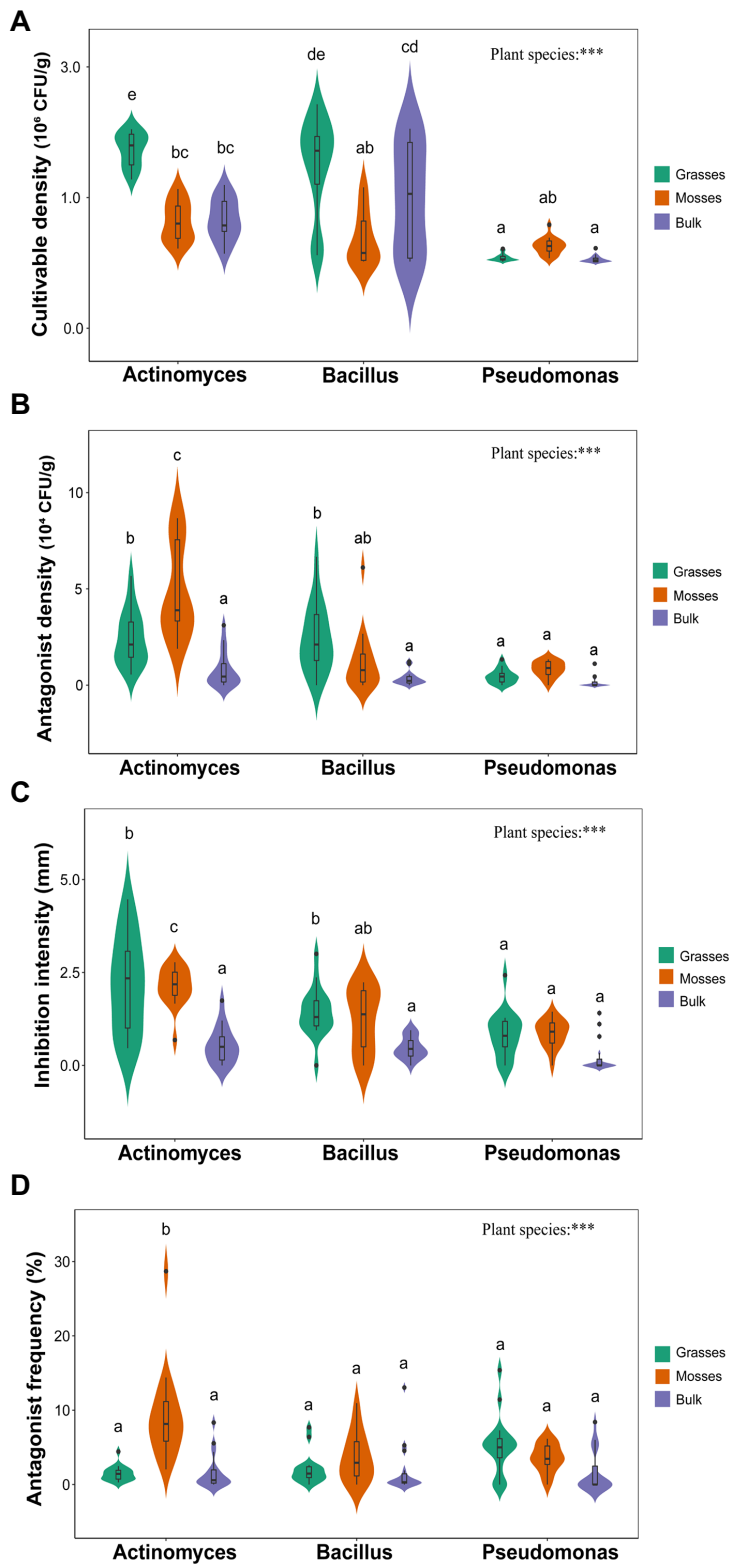
Overview of the effects of dominant plants on *Actinomyces*, *Bacillus*, and *Pseudomonas* antagonistic interactions **(A)** Total cultivable density of bacteria **(B)** the antagonistic density of bacteria **(C)** the radius of inhibition zone **(D)** the proportion of inhibitory *Actinomyces*, *Bacillus* and *Pseudomonas*. Abbreviations: Letters (a,b,c,…) above each bar represent the significance among genera (Tukey’s HSD). P′ indicates significant effects at ***p < 0.001.

*Actinomyces*, *Bacillus*, and *Pseudomonas* densities and antagonistic activities differed between vegetated and bulk soils. Among all experimental treatments, the highest total cultivable *Actinomyces* densities were recorded in grass-associated soils with a mean value of 1.7 × 10^6^ CFU/g against 0.66 × 10^6^ CFU/g in bulk soils (Figure 1A). However, the highest density of antagonistic *Actinomyces* was found in moss-associated soil with a mean value of 4.9 × 10^4^ CFU/g. In contrast, a mean of 0.8 × 10^4^ CFU/g of antagonistic *Actinomyces* was recorded in bulk soils. The number of antagonistic *Actinomyces* was increased by a factor of 10 when comparing bulk soil to vegetated soil (Figure 1B). Against the target strain, the average radius of inhibition zones, which measures the degree of inhibition, increased by a proportion of 4 and 1.5 among samples in vegetated and bulk soil, respectively. A considerable rise in inhibitory potential was observed in vegetated soil (Figure 1C). The frequency of antagonists (proportion) varied significantly among samples (Figure 1D). The highest cultivable densities of *Bacillus* were observed in grass-associated soils with a mean value of 1.4 × 10^6^ CFU/g, compared to 0.99 × 10^6^ CFU/g in bulk soil (Figure 1A). The highest antagonist densities were also found in grass-associated soils but were followed by moss-associated soils. Bulk soils only harbored 3,000 CFUs of antagonists per gram of soil on average (Figure 1B). The data revealed a significant increase in *Bacillus* antagonists and a considerable rise in inhibitory intensity when comparing bulk soil to vegetated soil (Figures 1B,C). Contrary to the inhibitory potential, the frequency of *Bacillus* antagonists did not significantly differ between samples (Figure 1D). Moss-associated soils presented the highest densities of *Pseudomonas* with a mean of 0.25 × 10^6^ CFU/g of soil (Figure 1A). *Pseudomonas* antagonists, frequency and inhibition potential showed little variation between samples (Figures 1B–D).

Overall, Antarctic dominant plants significantly influenced all the four indices of bacterial antagonistic interactions we measured. Specifically, total cultivable bacterial densities and antagonist densities differed significantly between vegetated and bulk soils. Grasses favored higher cultivable densities of *Actinomyces* and *Bacillus*. Whereas mosses supported higher *Pseudomonas* densities (Figure 1A). However, higher antagonist densities of *Actinomyces* and *Pseudomonas* were supported by mosses, whereas grasses supported higher *Bacillus* antagonists compared to bulk soils (Figure 1B).

The inhibition intensity of *Actinomyces* was higher in the vegetated soils than in bulk soils (Figure 1C). In parallel, *Bacillus* inhibitory intensity was significantly higher in grass-associated soils. However, inhibition intensities of the bacterial communities in bulk soils were not significantly different. Meanwhile, *Pseudomonas* inhibition potential significantly differed from *Bacillus* and *Actinomyces* in grass-associated soils (Figure 1C). Bacterial antagonist frequency and inhibition intensity also differed among soils. The moss-associated soils significantly favored the highest proportions (%) of *Actinomyces* compared to *Bacillus* and *Pseudomonas* (Figure 1D). Antagonist frequency of *Pseudomonas* was higher in grass-associated soils, whereas *Bacillus* antagonist proportion was higher in moss-associated soils in contrast to bulk soils (Figure 1D). Dominant plants had a strong association with all the indices of bacterial antagonistic interactions as compared to bulk soils (Supplementary Figure S3).

### The relationships between dominant plants, bacterial diversity and edaphic properties

**Figure 2:**
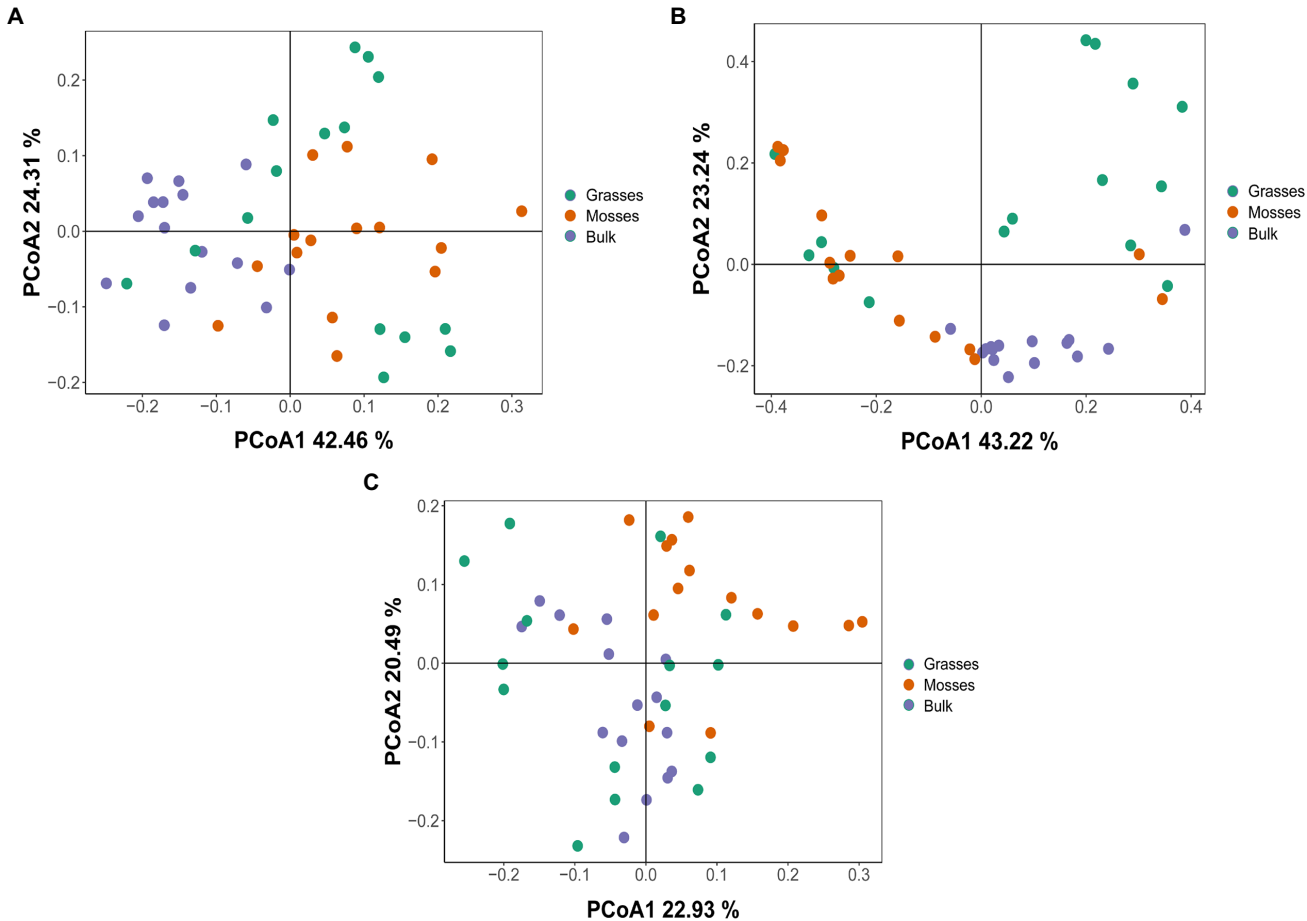
PCoA (Principal coordinate analysis) with Bray–Curtis dissimilarity to quantify compositional differences between samples based on bacterial OTUs at the genus level. PCoA1 (Axis 1) and PCoA2 (Axis 2) respectively explain the dissimilarity between samples **(A)** *Actinobacteria* (42.46 and 24.31%), **(B)** *Firmicutes* (43.22 and 23.24%) and **(C)** Proteobacteria (22.93 and 20.49%) across grasses, mosses and bulk soils.

Using the 16S rRNA data, we investigated the differences in bacterial communities of the three phyla of interest between vegetated and bulk soils. The PCoA did not identify clear clusters of unique bacterial communities. Overall, all soils (vegetated or not) shared a large number of *Actinobacteria* (Figure 2A), *Firmicutes* (Figure 2B) and Proteobacteria (Figure 2C). However, bulk soils did cluster closely for the *Firmicutes*, indicating some unique communities, compared to vegetated soils.

**Figure 3:**
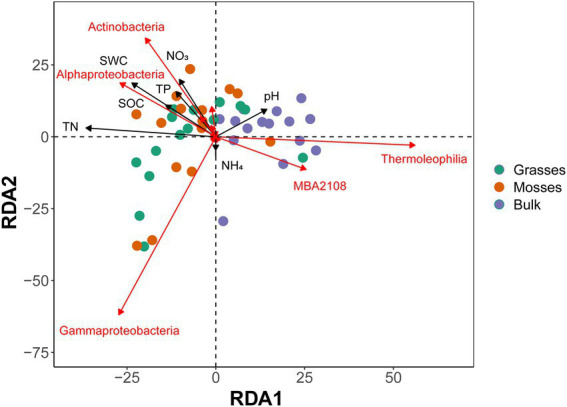
RDA (redundancy analysis) plot showing the effects of dominant plants and edaphic properties on the bacterial community at the class level from grasses, mosses and bulk soils. Abbreviations: SWC, soil water content; SOC, soil organic carbon; TN, total nitrogen; TP, total phosphorus; NO_3_^−^, nitrates; NH_4_^+^, ammonium. Only the bacterial classes with a large contribution to the first two axes are shown.

The redundancy analysis better discriminated the communities between treatments (Figure 3), highlighting more *Actinobacteria*, *Alphaproteobacteria* and *Gammaproteobacteria* in vegetated soils. The RDA also showed the significant influence of dominant plants on edaphic characteristics. Soil water content, soil organic carbon, total nitrogen, ammonium, and pH were significantly influenced by dominant plant species (Figure 3). Vegetated soils maintained a significant amount of soil resources when compared to bulk soils. The presence of mosses significantly affected soil water content and ammonium concentration in the associated soil. Similarly, grasses significantly affected organic carbon and total nitrogen in the rhizosphere (Supplementary Table S2).

To go further, a Pearson correlation analysis was conducted to evaluate the correlation between edaphic properties, antagonism indices and the relative abundance of the bacterial community (Supplementary Figure S4). It showed that densities and inhibition intensity of *Actinomyces*, antagonist density and inhibition intensity of *Bacillus*, and antagonist density, proportion, and intensity of *Pseudomonas* were positively correlated with the relative abundance of *Actinobacteria*. Likewise, soil water content and soil nitrates were strongly correlated with the soil *Actinobacteria* community. Similarly, the relative abundance of *Bacilli* was positively correlated with *Actinomyces* and *Bacillus* inhibition intensities. Furthermore, soil water content, soil organic carbon, total nitrogen, total phosphorus, and soil nitrates also positively correlated with the *Bacillus* community. Conversely, most of the studied soil properties were weakly correlated with *Gammaproteobacteria*. However, antagonist density and proportion of *Actinomyces* and density of *Pseudomonas* were positively correlated with the relative abundance of *Gammaproteobacteria*. Moreover, the relative abundances of other bacterial communities affiliated to the *Actinobacteria* (*Actinobacteria*, *Acidimicrobiia*, *Coriobacteriia*, *Rubrobacteria*, *Thermoleophilia*, MB-A2-108), *Firmicutes* (Clostridia, Desulfitobacteriia, Natranaerobiia, Negativicutes, BRH-c20a), and Proteobacteria (*Alphaproteobacteria*) also had a strong correlations with indices of antagonistic interactions and edaphic properties (Supplementary Figure S4).

### Direct and indirect effects of plants on bacterial antagonistic interactions

**Figure 4:**
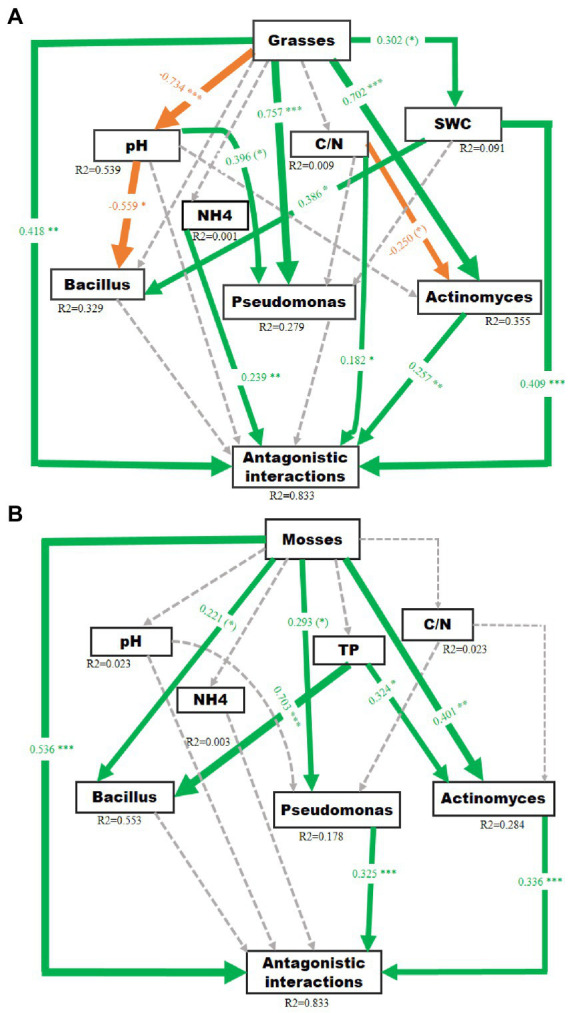
Structural equation models reflecting the direct and indirect (through changes in soil pH, carbon/nitrogen ratio, total phosphorus, soil water content and bacterial abundance) effects of Antarctic dominant plants on antagonistic potential in the soil of **(A)** Grasses P (Chi-square) = 0.50, P (RMSEA) = 0.57, (GFI) = 0.97 and **(B)** Mosses P (Chi-square) = 0.12, P (RMSEA) = 0.18, (GFI) = 0.90. Square boxes display variables included in the model: Exogenous variable (plant’s functional group affiliation) is given on top, endogenous variables below. Green and orange solid arrows indicate significant positive and negative effects, respectively. Dashed arrows indicate non-significant effects. Arrow width corresponds directly to the standardized path coefficient. R2 values associated with response variables indicate the proportion of explained variation by relationships with other variables. Values associated with arrows represent standardized path co-efficient. (*): *p* < 0.1, *p < 0.05, **p < 0.01, ***p < 0.001.

The SEM models explained 83.3% of the variation in antagonistic potential in the grass-associated soil (Figure 4A), and 83.3% variation in moss-associated soil (Figure 4B). The SEMs showed that both grasses and mosses had a direct significant effect on the bacterial antagonistic interactions. Furthermore, grasses strongly and significantly impacted the *Actinomyces* and *Pseudomonas* abundance. The *Bacillus* abundance and soil water content were marginally influenced by grasses and the pH of the soil, respectively. The grasses had no direct effect on carbon/nitrogen ratio and ammonium concentration. On the other hand, mosses had a direct significant influence on *Actinomyces* abundance and a marginal significant influence on *Bacillus* and *Pseudomonas* abundance. Soil phosphorus also had a significant influence on *Bacillus* and *Actinomyces* abundance, whereas mosses had no direct impact on phosphorus, ammonium, carbon/nitrogen ratio and pH. However, abundances of *Actinomyces* and *Pseudomonas* significantly influenced the antagonistic interactions.

## Discussion

Grasses and mosses may have different strategies to suppress pathogens and foster antagonistic bacteria. In this study, we employed a cultivation technique to assess bacterial antagonistic activity from Antarctic soils to determine the influence of dominant plant species on associated antagonists. However, we should note that as antibiotic synthesis is occasionally controlled in coordination with tissue development, this method may not discover the full potential for antagonism (Bakker et al., 2010). Our study is strongly supported by the recent research by Wang et al. (2022), which showed that plant species can impact microbial antagonistic potential. Our results showed that the Antarctic grass *Deschampsia antarctica* and Antarctic moss *Sanionia uncinata* had a significant direct influence on *Actinomyces*, *Bacillus*, and *Pseudomonas* antagonistic interactions and indirect influence *via* changes in edaphic properties and bacterial abundance.

The highest densities of *Actinomyces* and *Bacillus* communities were supported by grass-associated soil. This finding is consistent with other studies where major keystone taxa found in the rhizosphere of vascular plants in Antarctica were *Actinobacteria*, *Firmicutes*, and *Proteobacteria* (Zhang et al., 2020). However, the highest antagonistic densities and frequencies were favored by moss-associated soils. Indeed, the highest *Pseudomonas* densities, antagonistic densities, and frequencies were supported by moss-associated soils. This finding is consistent with studies showing that *Pseudomonas* is a well-known antagonistic genus in mosses (Berg, 2000; Lugtenberg et al., 2001). Overall, total densities, antagonistic densities, and inhibition intensity of bacterial communities were highest in the vegetated soils of terrestrial Antarctica. This finding suggests that plants supported by more antagonists favors more suppressive soil and it is supported by several other studies which showed that more antagonists leads to more suppression (de Souza et al., 2003; Bressan and Figueiredo, 2008).

### Direct and indirect effect of grasses on *Actinomyces*, *Bacillus* and *Pseudomonas* antagonistic interactions

The presence of grasses increased the abundance of *Actinomyces* and *Pseudomonas* while somewhat reducing the abundance of *Bacillus* and negatively affecting the pH of the soil. Grasses positively impacted *Pseudomonas* and *Actinomyces* abundance by increasing soil moisture level, increasing the antagonistic interactions. The outcome is in line with earlier studies, where the presence of *Pseudomonas* with genes associated with the production of antifungal compounds was positively impacted by grasses (Latz et al., 2012). However, this result contrasts with other studies, where grasses decreased *Actinomyces* and *Pseudomonas* abundance (Latz et al., 2016).

The *Actinomyces* abundance and ammonium concentration, in turn, directly increased the antagonistic interactions. Grasses also had a direct positive effect on soil moisture. Moreover, the carbon/nitrogen ratio in the soil had a marginal negative influence on *Actinomyces* abundance; however, it increased antagonistic interactions directly and indirectly *via Actinomyces* abundance. In contrast, an increase in *Actinomyces* abundance and soil moisture was associated with bacterial antagonistic interactions. An enhancement in suppression in natural ecosystem is frequently correlated with an increase in antagonistic or competitive activity in one or more soil microbial community components (Garbeva et al., 2004; Janvier et al., 2007). Meanwhile, we found significant associations between measures of *Actinomyces*, *Bacillus,* and *Pseudomonas* antagonistic interactions and the edaphic characteristics of the soil indicating that alterations in the chemical environment of the soil may serve as a partial mediator of plant-driven impacts on related bacterial communities (Fierer and Jackson, 2006).

### Direct and indirect effect of moss species on *Actinomyces*, *Bacillus* and *Pseudomonas* antagonistic interactions

Mosses increased *Actinomyces* and *Pseudomonas* abundance, increasing antagonistic interactions by decreasing the pH in the soil. Mosses directly increased the abundance of *Bacillus*, whereas grasses had no direct impact on *Bacillus* abundance. Mosses had no direct influence on soil phosphorus, however; it strongly affected the abundance of *Actinomyces* and *Bacillus* and increased the antagonistic interactions *via* the increase of *Actinomyces* communities in the soil. Therefore, the effects of Antarctic dominant plants seem to be species-specific, as previously suggested (Latz et al., 2015). Research showed that the effects of mosses on microbial communities are mostly associated with soil properties and soil nutrients (Bao et al., 2019), and that mosses increase the stability of the soil (Belnap and Büdel, 2016), with more stable soils being considered more fertile and harboring more abundant nutrients (Belnap and Eldridge, 2001; Valentin, 2002). This is supported by our results which indicate that vegetated soils harbored higher nutrient availability compared to bulk soils (Supplementary Table S2).

Overall, our results suggest that bacterial community composition, edaphic properties and nutrient availability influence the antagonistic interactions of bacterial communities in soil. We also found that antagonistic interactions of *Actinomyces*, *Bacillus* and *Pseudomonas* were strongly influenced by Antarctic dominant plants and mediated by edaphic properties and bacterial abundance. This outcome is in line with the findings that Antarctic plant species are associated with nutrient cycling, microbial abundance and soil functioning (Benavent-González et al., 2018).

## Conclusion

Our research provides a better understanding of the direct and indirect effect of Antarctic dominant plants on soil bacterial antagonistic interactions. Antagonistic *Actinomyces*, *Bacillus*, and *Pseudomonas* contribute significantly to plant–soil feedbacks. Moreover, soil physicochemical properties play an important role in modulating the effect of Antarctic plants on bacterial antagonistic interactions in soil. We concluded that vegetated soils are more functional and competitive than bulk soils, suggesting that Antarctic dominant plants significantly influence bacterial activity in the soil and plant-associated soil favor more antagonistic bacterial communities than bulk soil. This study provides a theoretical basis for studying the important factors such as dominant plants, soil physicochemical properties and bacterial community composition regulating bacterial interactions in Antarctic soil.

## Data availability statement

The datasets presented in this study can be found in online repositories. The names of the repository/repositories and accession number(s) can be found below: NCBI - PRJNA921735.

## Author contributions

SC and BN designed the study and performed laboratory experiments. IzA, HS, YW, XL, MU, IkA, SX, and KL performed field experiments. BN, ZL, and SX performed statistical analysis and interpretation of data. BN and LM drafted the manuscript. LA, SX and SC provided scientific research funds. All authors contributed to the article and approved the submitted version.

## Funding

This research was supported by the Project of the National Natural Science Foundation of China (41830321, 31870412, 32071532). This research was also supported by Second Tibetan Plateau Scientific Expedition and Research (STEP) Program (2019QZKK0302).

## Conflict of interest

The authors declare that the research was conducted in the absence of any commercial or financial relationships that could be construed as a potential conflict of interest.

## Publisher’s note

All claims expressed in this article are solely those of the authors and do not necessarily represent those of their affiliated organizations, or those of the publisher, the editors and the reviewers. Any product that may be evaluated in this article, or claim that may be made by its manufacturer, is not guaranteed or endorsed by the publisher.
