# Supplementary material for: Dominant plant species play an important role in regulating bacterial antagonism in terrestrial Antarctica
Source: Front Microbiol. 2023 Mar 23;14:1130321. doi: 10.3389/fmicb.2023.1130321 (PMC10076557; doi:10.3389/fmicb.2023.1130321)
Supplement: Supplementary file 1 [file Data_Sheet_1.pdf]

Supplementary data

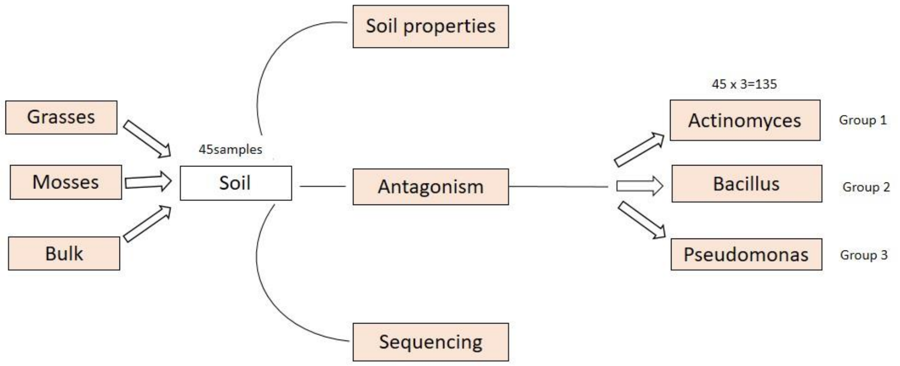

FIGURE S1 Flow chart for soil distribution (n=135)

Table S1 Hypothesis liable for the structural equation model (Pathways referred to Figure S2).

Number	Reference
**Pathway 1**	(Mendes et al. 2011), (Latz et al. 2016), (Latz et al. 2012)
**Pathway 2**	(Janvier et al. 2007)
**Pathway 4-3**	(Bueno et al. 2016), (Benavent-González et al. 2018)
**Pathway 4-6**	(Tian and Li 2017), (Opelt and Berg 2004)
**Pathway 5-3**	(Latz et al. 2016), (Lange et al. 2014), (Pérès et al. 2013)
**Pathway 5-6**	(Latz et al. 2012), (Eisenhauer et al. 2010), (Latz et al. 2016)

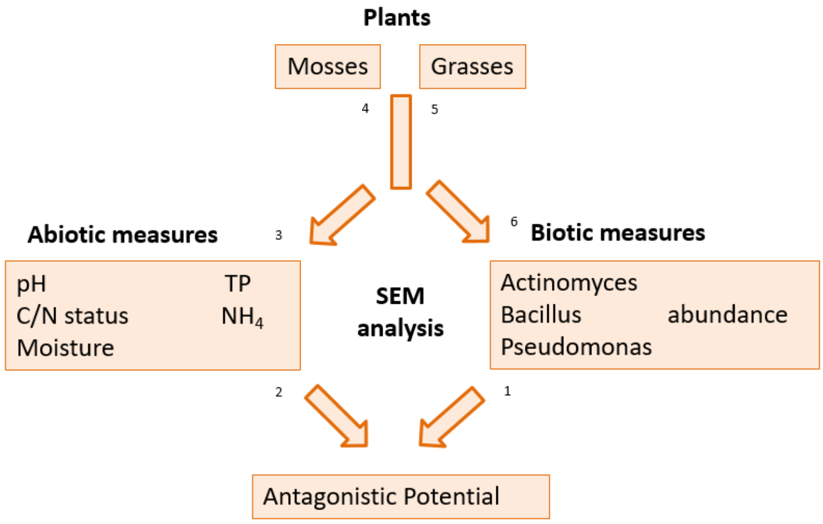

FIGURE S2 The conceptual figure of structural equation models shows that plant community composition may affect bacterial antagonistic potential via soil abiotic and biotic measures. Numbers (1,2,3…) near each arrow represent pathways. Abbreviations: C/N, carbon/nitrogen ratio; TP, total phosphorus; NH_4^+, ammonium; SEM, structure equation modeling

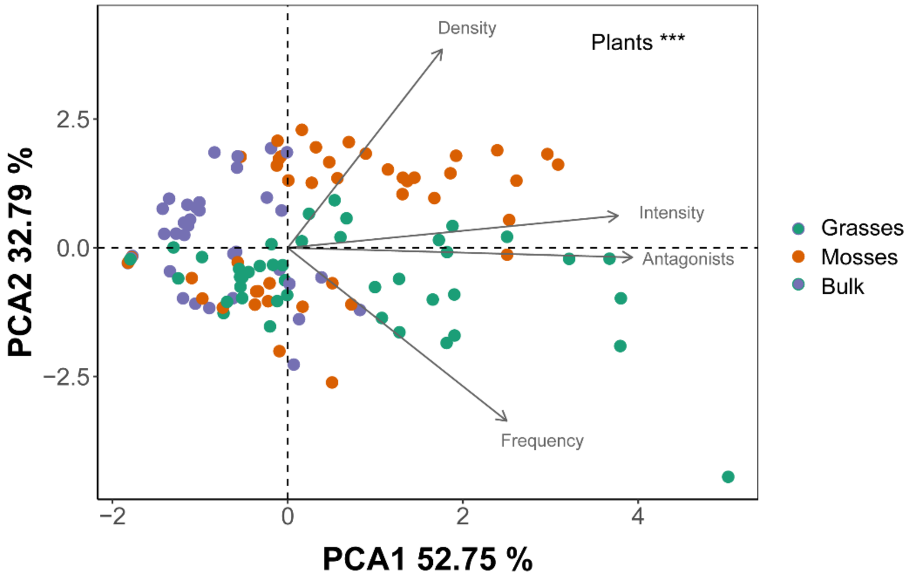

FIGURE S3 Principal component analysis (PCA) using PERMANOVA showing dominant plants effects on combined bacterial antagonism (n=135).

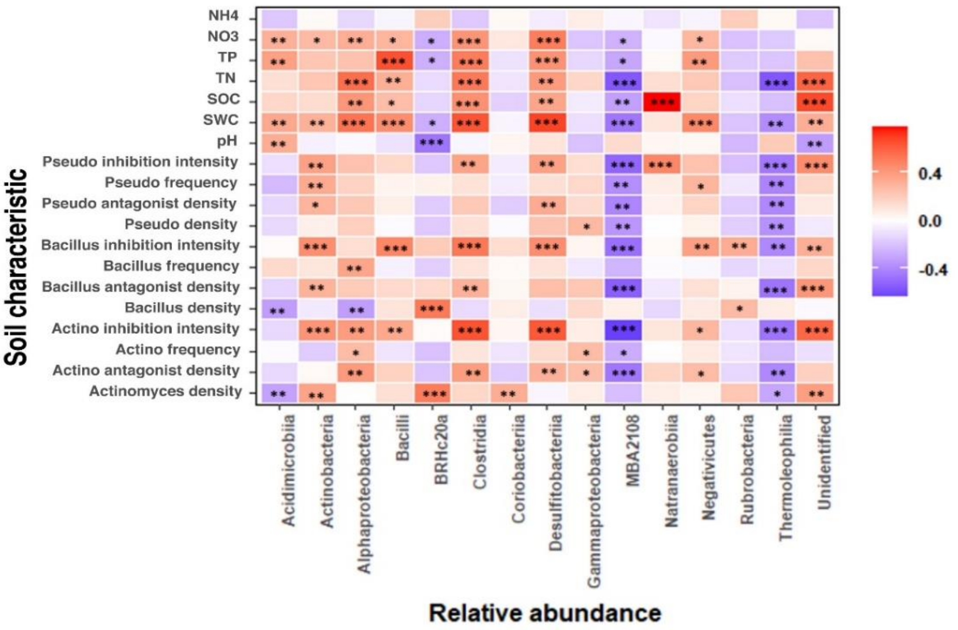

FIGURE S4 Heatmap with Pearson correlation showing the relationship between bacterial relative abundance at the class level and soil characteristics and indices of bacterial antagonism (* P< 0.1, **P< 0.05, *** P< 0.001)

Table S2 Overview of dominant plant effects on soil edaphic properties (mean ± SE). The boldface means significant effects of dominant plants (P<0.05). Abbreviation: SWC, moisture; SOC, organic carbon, TN, total nitrogen; TP, total phosphorus; NO_3^-, nitrates; NH_4^+, ammonium

	Bulk	Mosses	Grasses	P-value
**Edaphic properties**				
**SWC (%)**	**0.17±0.04**	**0.23±0.11**	**0.20±0.06**	**0.0016**
**SOC (mg/g)**	**8.80±4.57**	**15.7±8.33**	**19.2±19.9**	**0.0378**
**TN (mg/g)**	**1.05±0.41**	**1.97±1.00**	**2.29±1.27**	**< 0.001**
**TP (mg/g)**	0.76±0.26	0.77±0.24	0.74±0.16	0.8738
**NO_3 (mg/g)**	41.3±19.4	48.2±27.9	33.5±17.7	0.1207
**NH_4 (mg/g)**	**14.2±7.80**	**14.9±4.96**	**14.5±4.87**	**< 0.001**
**pH**	**7.41±0.12**	**7.38±0.17**	**7.11±0.16**	**< 0.001**

References

Benavent-González A, Delgado-Baquerizo M, Fernández-Brun L, et al (2018) Identity of plant, lichen and moss species connects with microbial abundance and soil functioning in maritime Antarctica. Plant Soil 429:35–52. https://doi.org/10.1007/s11104-018-3721-7

Bueno CG, Williamson SN, Barrio IC, et al (2016) Moss Mediates the Influence of Shrub Species on Soil Properties and Processes in Alpine Tundra. PLoS One 11:e0164143. https://doi.org/10.1371/journal.pone.0164143

Eisenhauer N, Beßler H, Engels C, et al (2010) Plant diversity effects on soil microorganisms support the singular hypothesis. Ecology 91:485–496. https://doi.org/10.1890/08-2338.1

Janvier C, Villeneuve F, Alabouvette C, et al (2007) Soil health through soil disease suppression: Which strategy from descriptors to indicators? Soil Biol Biochem 39:1–23. https://doi.org/10.1016/j.soilbio.2006.07.001

Lange M, Habekost M, Eisenhauer N, et al (2014) Biotic and abiotic properties mediating plant diversity effects on soil microbial communities in an experimental grassland. PLoS One 9:e96182. https://doi.org/10.1371/journal.pone.0096182

Latz E, Eisenhauer N, Rall BC, et al (2012) Plant diversity improves protection against soil-borne pathogens by fostering antagonistic bacterial communities. J Ecol 100:597–604. https://doi.org/10.1111/j.1365-2745.2011.01940.x

Latz E, Eisenhauer N, Rall BC, et al (2016) Unravelling Linkages between Plant Community Composition and the Pathogen-Suppressive Potential of Soils. Sci Rep 6:23584. https://doi.org/10.1038/srep23584

Mendes R, Kruijt M, De Bruijn I, et al (2011) Deciphering the rhizosphere microbiome for disease-suppressive bacteria. Science (80-) 332:1097–1100. https://doi.org/10.1126/SCIENCE.1203980/SUPPL_FILE/PAP.PDF

Opelt K, Berg G (2004) Diversity and antagonistic potential of bacteria associated with bryophytes from nutrient-poor habitats of the baltic sea coast. Appl Environ Microbiol 70:6569–6579. https://doi.org/10.1128/AEM.70.11.6569-6579.2004

Pérès G, Cluzeau D, Menasseri S, et al (2013) Mechanisms linking plant community properties to soil aggregate stability in an experimental grassland plant diversity gradient. Plant Soil 373:285–299. https://doi.org/10.1007/s11104-013-1791-0

Tian Y, Li YH (2017) Comparative analysis of bacteria associated with different mosses by 16S rRNA and 16S rDNA sequencing. J Basic Microbiol 57:57–67. https://doi.org/10.1002/jobm.201600358
